# Apocrine secretion by the choroid plexus

**DOI:** 10.1186/s12987-025-00684-3

**Published:** 2025-07-16

**Authors:** Ya’el Courtney, Maria K. Lehtinen

**Affiliations:** 1 Department of Pathology, Boston Children’s Hospital, Harvard Medical School, Boston, MA 02115 USA; 2 Graduate Program in Neuroscience, Harvard Medical School, Boston, MA 02115 USA

**Keywords:** Apocrine secretion, Choroid plexus, Cerebrospinal fluid, Epithelial cells, Exocytosis, Calcium, Neuromodulation, Extracellular vesicles

## Abstract

The choroid plexus (ChP) epithelium secretes cerebrospinal fluid (CSF) and signaling factors that influence brain development. In addition to classical secretory pathways, the ChP also employs apocrine secretion, in which large cytoplasmic portions bud from the apical surface in structures called aposomes. Although historically underappreciated, recent imaging and molecular studies demonstrate that this process is calcium-dependent and regulated by neuromodulators such as serotonin. Apocrine secretion contributes distinct cytoplasmic cargo—proteins, organelles, and signaling molecules—to the CSF, with evidence for developmental roles in neurogenesis and progenitor cell differentiation. This review synthesizes structural, functional, and proteomic data supporting ChP apocrine secretion, compares it to other epithelial release mechanisms, and highlights outstanding questions about its regulation and physiological roles. By focusing on this unconventional and understudied mode of secretion, we provide a framework for understanding how ChP-mediated cargo release shapes the CSF environment and contributes to brain development.

## Background

**Fig. 1:**
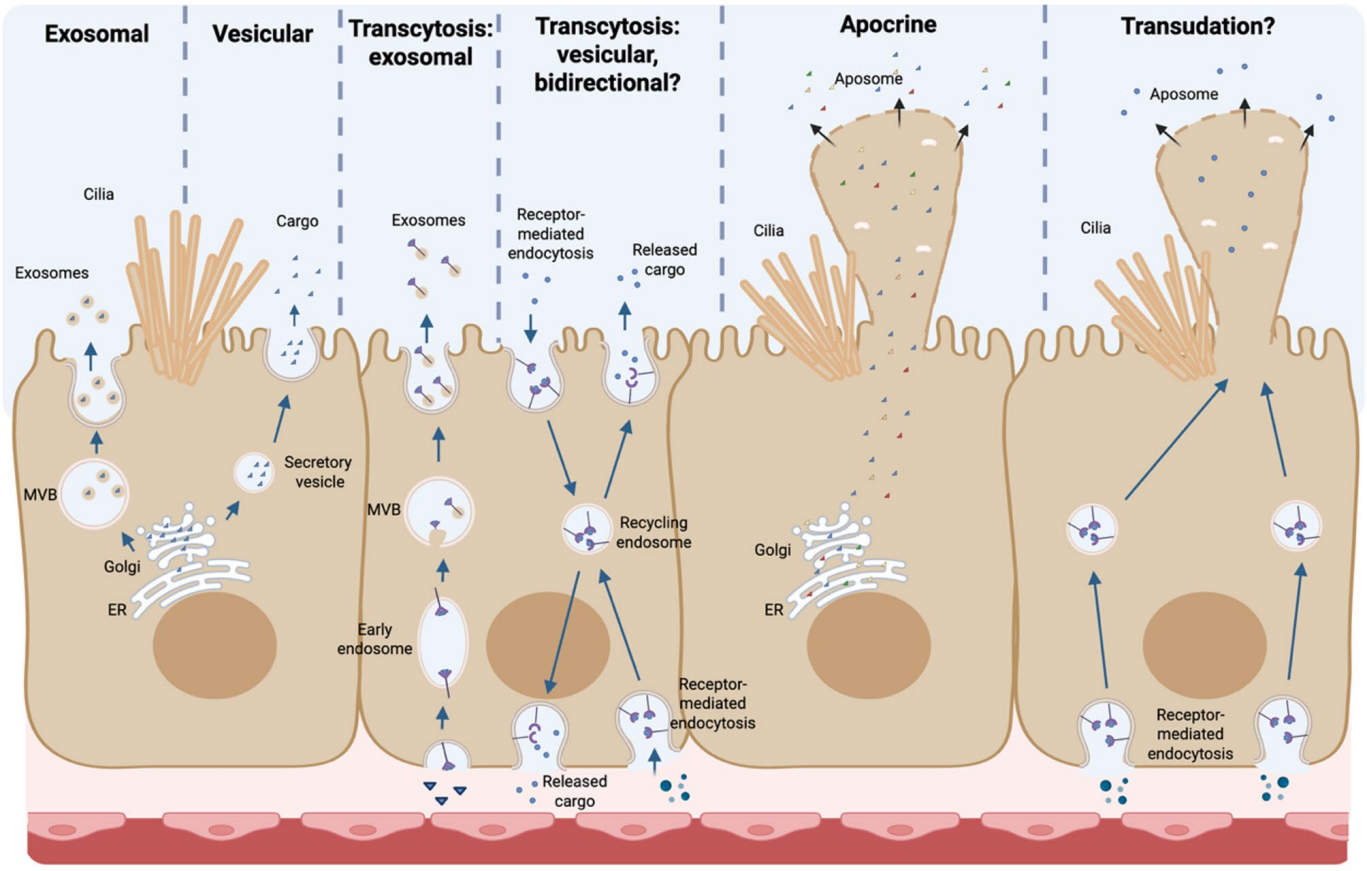
Modes of Demonstrated and Hypothesized ChP Exocytosis. This figure illustrates the diverse mechanisms by which ChP epithelial cells may release cargo into the CSF. The pathways include exosomal release via multivesicular bodies (MVBs), vesicular secretion through direct fusion with the plasma membrane, and transcytosis in both exosomal and vesicular forms, allowing cargo transport across the cell. Apocrine secretion involves the budding and release of membrane-enclosed cytoplasmic portions (aposomes) from the apical surface. Transudation has also been hypothesized as a possible mechanism for rapid paracellular passage or fluid exchange, although its existence in the ChP remains unconfirmed

The choroid plexus (ChP) critically influences neural development and homeostasis through its role in cerebrospinal fluid (CSF) production and composition [1–3]. While classical secretory mechanisms such as exocytosis and transcytosis have long been recognized [4–7], the apocrine secretion pathway [8–11], characterized by the apical budding of cytoplasmic material in structures called aposomes, is emerging as a significant contributor to the CSF proteome. Initially described decades ago [12–16], aposomes were historically dismissed as artifacts of fixation or cellular damage [17, 18]; however, recent technological advances have confirmed their physiological relevance, particularly calcium dependence and neuromodulatory regulation via the serotonin receptor 5HT2C [19, 20]. Table 1 provides a summary of secretion modes demonstrated in the ChP, illustrated visually in Fig. 1.

**Table 1:** Summary of choroid plexus (ChP) exocytosis modes and the experimental models used to study them. Structural evidence (*) and functional evidence (†) are indicated for each model. References supporting the findings are provided in the brackets

Mode of ChP Exocytosis	Model Used [Supporting Literature] *Structural only, ^†^Functional
Exosome/extracellular vesicle release	Human^†^ [21] Human–in vitro–HCPEpiC^†^ [21] Mouse^†^ [4, 22, 23] Mouse–primary culture^†^ [4, 24] Rat* [25]
Vesicle fusion	Mouse–ex vivo^†^ [26]
Transcytosis: exosomal	Rat–in vitro–Z310 cell line^†^ [7]
Transcytosis: vesicular; basal to apical	Mouse^†^ [27–29] Mouse–primary culture^†^ [30] Rat^†^ [31, 32]
Transcytosis: vesicular; apical to basal	Mouse^†^ [27] Mouse–partial^†^ [28] (*this study supports endocytosis from the apical side but not re-release on the basal side*) Mouse–primary culture^†^ [33] Rat–primary culture^†^
Apocrine	Buffalo* [34] Cat*^†^ [35, 36] Dog*^†^ [12, 37] Frog* [12] Gerbil* [12] Goat* [38] Human* [39–43] Human–primary culture*^†^ [20, 44, 45] Monkey* [12, 35, 46, 47] Mouse*^†^ [20, 26, 48] Pig* [49] Rabbit* ^12,50^ Rat*^†^ [12, 13, 16, 51–64] Turtle* [12, 65]
Transudation	No primary evidence, hypothesized in [9]

## Structural and functional evidence

Initial anatomical reports from the mid-20th century documented aposome-like structures in the ChP across various species [12, 35, 38, 43, 44, 50, 61], although these were often discounted due to methodological concerns. Aposomes are considerably larger than classical extracellular vesicles, typically ranging from 1 to 5 μm in diameter, compared to microvesicles (100–1000 nm) and exosomes (30–100 nm). Recent studies employing advanced real-time imaging techniques and proteomics have provided strong functional evidence for calcium-dependent apocrine secretion in the ChP [19, 20]. Live imaging of calcium-associated apical membrane release events, as shown in Shipley et al. [19], supports dynamic, non-apoptotic extrusion from the apical surface. These events involve extensive membrane remodeling and result in the release of cytoplasmic contents, including organelles and signaling proteins, without compromising cell viability [8, 12, 20]. Serotonin, among other neuromodulators, robustly induces intracellular Ca²⁺ elevations, triggering apocrine release [19, 20].

**Fig. 2:**
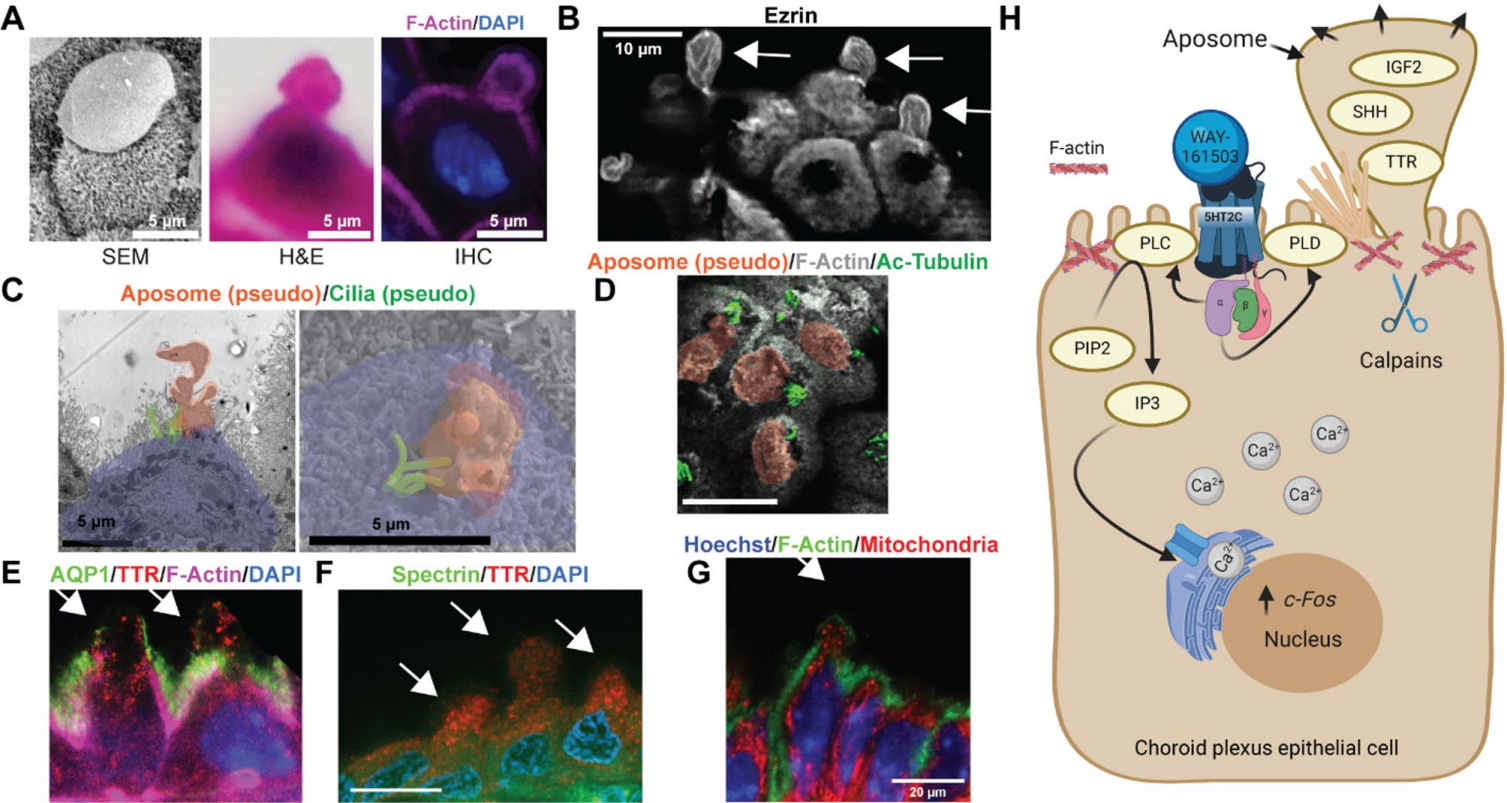
Structural and Functional Evidence for Apocrine Secretion in the Choroid Plexus. (**A**) Scanning electron micrograph (SEM), H&E staining, and immunofluorescence (IHC) images depicting apocrine structures (aposomes) budding from the apical surface of mouse ChP epithelial cells. (**B**) Immunofluorescence staining for Ezrin highlighting apical protrusions in mouse ChP epithelial cells. (**C-D**) Correlative microscopy revealing the close spatial association between mouse aposomes (pseudocolored) and cilia bundles, marked by pseudocolor or acetylated Tubulin staining, respectively. (**E**) Immunofluorescence images demonstrating apical enrichment of Aqp1 and TTR proteins in mouse ChP epithelial cells. (**F**) Spectrin immunofluorescence in human ChP epithelial cells. (**G**) Mitochondrial staining in mouse ChP epithelial cells; signal is distributed throughout the cytoplasm and not restricted to the apical domain. (**H**) Schematic model illustrating the proposed calcium-dependent signaling pathways and molecular effectors regulating ChP apocrine secretion, including serotonin receptor activation, phospholipase (PLC/PLD) activation, IP3 production, calcium release, and calpain activation. Figure adapted from Courtney et al. 2025 ^20^

Structural evidence from electron microscopy, immunofluorescence, and histology further validates the presence of aposomes and reveals their consistent spatial proximity to cilia (Fig. 2). This spatial proximity may reflect a coordinated apical architecture, or could suggest a functional relationship, such as a role for cilia in sensing or dispersing aposomal contents, though this remains speculative. Aposome-associated protrusions are enriched in apical cytoskeletal and membrane markers such as actin, ezrin, spectrin, and aquaporin-1. While not specific to aposomes, these markers highlight regions of active membrane remodeling characteristic of apocrine secretion.

## Aposome composition and physiological roles

Proteomic analysis has identified diverse aposomal contents, including growth factors, enzymes, and immune modulators [20]. Although aposomes are membrane-enclosed, their contents may include both membrane-bound proteins and soluble cytosolic material, consistent with the mixed composition observed in other apocrine systems. While lipids, regulatory RNAs, and structurally complex cargo have not yet been directly demonstrated in ChP aposomes, studies on apocrine secretion in other tissues suggest that such molecules could also be exported [8, 10, 11]. Recent evidence specifically indicates that apocrine secretion significantly influences embryonic brain development by regulating neural progenitor proliferation and differentiation. Importantly, disruptions in prenatal apocrine secretion due to maternal exposure to 5HT2C agonists or maternal immune activation (Poly I: C model) can alter fetal CSF composition, with potential implications for neurodevelopmental disorders [20].

## Open questions

Key unresolved questions remain about apocrine secretion, including the precise molecular machinery governing aposome formation and cargo selection, mechanisms that ensure membrane and cellular integrity post-secretion, and the identification of specific triggers and modulatory pathways. Understanding these aspects could lead to novel therapeutic strategies leveraging apocrine secretion for targeted neuroactive molecule delivery.

## Conclusion

Apocrine secretion in the ChP is emerging as a critical yet previously underappreciated mechanism for influencing the central nervous system environment. By enabling the bulk release of proteins, lipids, and other cytoplasmic components directly into the CSF, this secretory mode offers a powerful and adaptable means of modulating neural development, homeostasis, and, potentially, response to injury or disease. Although much remains to be learned about the molecular triggers, cargo recruitment mechanisms, and target-specific responses, recent imaging, molecular, and proteomic advances are beginning to clarify how ChP apocrine secretion complements more traditional exocytotic and transcytotic pathways. As we continue to uncover its cellular and molecular underpinnings, apocrine secretion may open new horizons in understanding brain biology and designing targeted therapeutic interventions.

## Data Availability

No datasets were generated or analysed during the current study.
